# Effects of Red Palm Oil on Myocardial Antioxidant Enzymes, Nitric Oxide Synthase and Heart Function in Spontaneously Hypertensive Rats

**DOI:** 10.3390/ijms18112476

**Published:** 2017-11-21

**Authors:** Emma Katengua-Thamahane, Barbara Szeiffova Bacova, Iveta Bernatova, Matus Sykora, Vladimir Knezl, Jacques Van Rooyen, Narcis Tribulova

**Affiliations:** 1 Botho University, Francistown Campus, Plot 6434 Tati River plots, Francistown P/Bag F451, Botswana; ekatengua@yahoo.co.uk; 2 Institute for Heart Research, Slovak Academy of Sciences, Dúbravská cesta 9, P.O. Box 104, 840 05 Bratislava, Slovakia; matus.sykora@savba.sk (M.S.); narcisa.tribulova@savba.sk (N.T.); 3 Institute of Normal and Pathological Physiology, Slovak Academy of Sciences, Sienkiewiczova 1, 813 71 Bratislava, Slovakia; iveta.bernatova@savba.sk; 4 Institute of Experimental Pharmacology and Toxicology, Slovak Academy of Sciences, Dúbravská Cesta 9, 841 04 Bratislava, Slovakia; vladimir.knezl@savba.sk; 5 Binutra, Unit E 11 Prime Business Park Mocke Rd., Diep River, Cape Town 7806, South Africa; jacques.vanrooyen@bio-cap.com

**Keywords:** red palm oil, hypertensive rats, antioxidant enzymes, heart function

## Abstract

The purpose of this study was to investigate the effect of antioxidants rich red palm oil (RPO) supplementation on cardiac oxidative stress known as crucial factor deteriorating heart function in hypertension. 3-month-old, male spontaneously hypertensive rats (SHR) and normotensive Wistar Kyoto rats (WKY) were fed standard rat chow without or with RPO (0.2 mL/day/5 weeks). General characteristic of rats were registered. Left ventricular tissue (LV) was used to determine expression of superoxide dismutases (SOD1, SOD2) and glutathione peroxidases (Gpx) as well as activity of nitric oxide synthase (NOS). Functional parameters of the heart were examined during basal conditions and at the early-phase of post-ischemic reperfusion using Langendorff-perfused system. RPO intake significantly reduced elevated blood pressure and total NOS activity as well as increased lowered expression of mitochondrial SOD2 in SHR hearts during basal condition. Moreover, RPO supplementation resulted in suppression of elevated heart rate, increase of reduced coronary flow and enhancement of systolic and diastolic heart function at the early-phase of post-ischemic reperfusion. It is concluded that SHR benefit from RPO intake due to decrease of blood pressure, amelioration of oxidative stress and protection of heart function that was deteriorated by post-ischemic reperfusion.

## 1. Introduction

Hypertension is an enormous public health burden, which poses significant cause of morbidity and mortality associated with cardiovascular diseases. It is also an important independent risk factor for cardiovascular diseases [1,2]. The pathophysiology of essential hypertension is complex and not fully elucidated. It may result from genetic and environmental factors [3,4,5] as well as from increased cardiovascular response to sympathetic nerve [6]. The hallmark of hypertension is elevation of blood pressure, which if untreated, could have serious impact on renal failure and increased incidence of heart failure [1,7]. Some of the most serious complications of hypertension include left ventricular remodelling (hypertrophy), endothelial dysfunction, compromised ventricular function and increased susceptibility of the myocardium to ventricular arrhythmias and sudden cardiac death [8]. Cardiac hypertrophy is one of the most deleterious consequences of hypertension and it has been shown to be an important predictor of mortality and sudden cardiac death in hypertensive heart disorders [8,9,10].

A growing body of scientific evidence suggests that oxidative stress may play a pivotal role in both the pathogenesis of hypertension and exacerbation of hypertensive heart disorders [3,8,10,11,12,13,14]. Increased production of reactive oxygen species (ROS), especially superoxide anion, in hypertension may lead to impairment of endogenous antioxidant defense systems and to decline of nitric oxide (NO) bioavailability [15,16]. Superoxide dismutases (SODs) are the major antioxidant defense systems against superoxide anion. They consist of three isoforms requiring catalytic metal (Cu or Mn) for their activation: the cytosolic Cu/ZnSOD (SOD1), the mitochondrial MnSOD (SOD2), and the extracellular Cu/ZnSOD (SOD3). SODs, in each subcellular location, catalyse the conversion of superoxide anion to H_2_O_2_, which may participate in cell signalling. H_2_O_2_ is reduced to H_2_O by catalase or glutathione peroxidases (GPx). SODs play a critical role in inhibiting oxidative inactivation of NO and preventing peroxynitrite formation, thereby endothelial and mitochondrial dysfunction [17,18].

It has been reported that antioxidant-rich red palm oil (RPO) exerts cardio-protection against ischaemia reperfusion injury [19,20,21]. It is probably due to its ratio of saturated/unsaturated fatty acid and high phytonutrients concentration. RPO contains 51% of saturated fatty acids, 38% of monounsaturated fatty acids and 11% of polyunsaturated fatty acids. This oil is also abundant on micronutrients such as carotenes (namely α- or β-carotene), vitamin E (namely tocopherols and tocotrienols), phytosterols, squalene and coenzyme Q10 [22,23].

One of the possible mechanisms underlying RPO-induced salutary effect could be via modulation of the nitric oxide/cyclic guanosine monophosphate (NO-cGMP) signalling pathway with a potential to increase nitric oxide bioavailability [24]. Szucs et al. [25] showed that RPO reduced myocardial infarct size in hearts of rats fed a high cholesterol diet (known to be accompanied by excess of ROS). This anti-ischemic effect was associated with attenuation of lipid disorders and reduced pre-ischaemic matrix metalloproteinase 2 activity. RPO supplementation was also beneficial against anthracycline-induced cardiac toxicity via attenuation of down-regulation of SOD1 [26]. It could be partly related to the antioxidant activity of RPO and its ability to modulate the oxidative sensitive mitogen-activated protein kinase (MAPK) signalling pathways. Our recent study [27] demonstrated that dietary RPO reduced blood pressure, blood glucose, post-ischemic reperfusion-induced arrhythmias, electrically inducible ventricular fibrillation (VF), and improved post-ischemic heart function in spontaneously hypertensive rats (SHR). The anti-arrhythmic effect was linked to up-regulation of myocardial electrical coupling protein connexin 43 that is impaired in conditions of oxidative stress [8]. Significant reduction of blood pressure in SHR due to palm oils intake was observed by others as well [28].

Taken together, the beneficial effect of RPO on cardiovascular health may involve its ability to improve endogenous antioxidant defense mechanisms and modulation of the redox sensitive signalling pathways. In view of this evidence, the aim of this study was to explore whether the specific antioxidant enzymes (SOD1, SOD2 and Gpx1) as well as the NO-cGMP pathway are involved in RPO induced heart protection in SHR. It may contribute to better understanding of the underlying mechanisms of RPO-induced cardio-protection in SHR, a model of human essential hypertension.

## 2. Results

### 2.1. Main Characteristics of Experimental Rats

Compared to normotensive Wistar Kyoto rats (WKY), male hypertensive rats exhibited significantly higher systolic blood pressure as well as left ventricular weight. Body weight of hypertensive rats was lower comparing to age-matched WKY. Consumption of RPO significantly reduced blood pressure in hypertensive rats. Data are summarized in Table 1.

### 2.2. Effect of Dietary Red Palm Oil (RPO) on the Myocardial SOD1 and SOD2 Protein Expression in Normotensive and Hypertensive Rats

**Figure 1:**
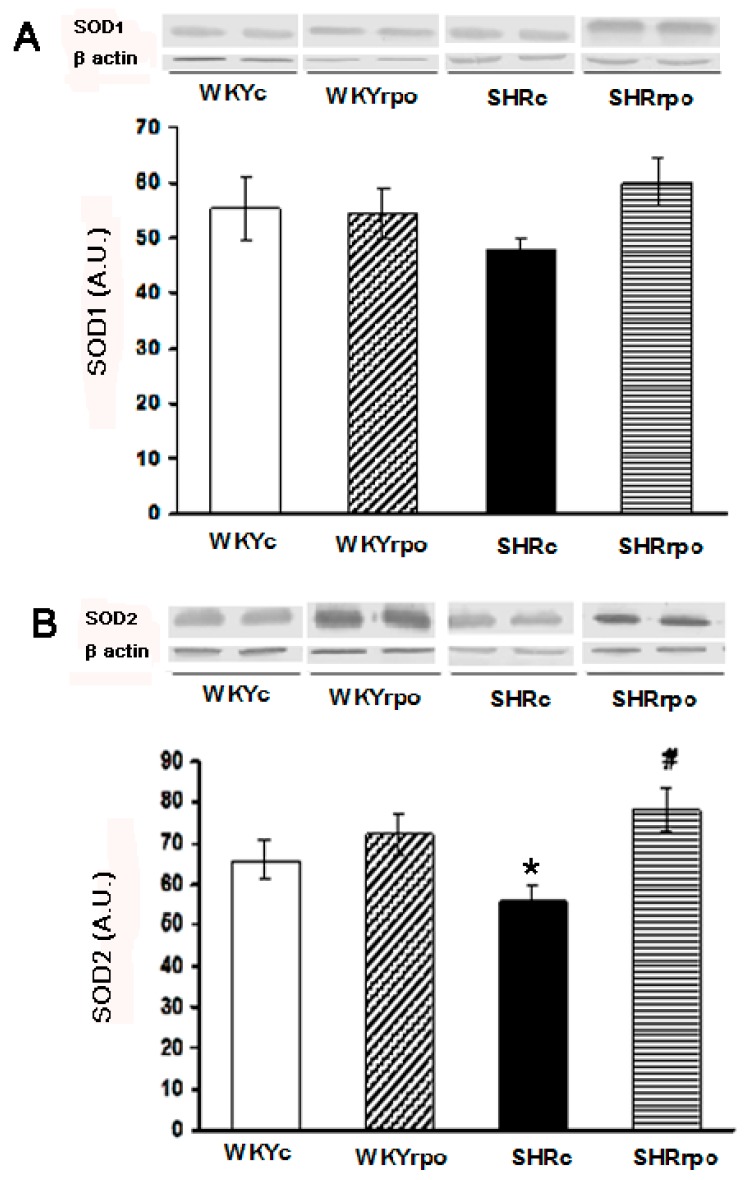
Effect of dietary red palm oil (RPO) supplementation on the protein expression of superoxide dismutase 1 (SOD1. (**A**)) and superoxide dismutase 2; (SOD2. (**B**)) in the left ventricle of normotensive and hypertensive rats. WKYc—Wistar Kyoto control rats; WKYrpo—WKY treated with RPO; SHRc—Spontaneously hypertensive rats; SHRrpo—SHR treated with RPO; Results are expressed as mean ± SEM; *n* = 5 in each group; * *p* < 0.05 versus control rats (WKYc); ^#^ *p* < 0.05 WKY/SHR versus treated with RPO; A.U.—Arbitrary units.

Compared to normotensive WKY, myocardial expression of SOD2 was significantly decreased in SHR hearts (Figure 1B). RPO supplementation significantly increased mitochondrial SOD2 levels in SHR (Figure 1B). There were no significant differences in SOD1 and SOD2 proteins expression in WKY supplemented with RPO (Figure 1A,B).

### 2.3. Effect of Dietary RPO on the Myocardial Gpx1 Protein Expression in Normotensive and Hypertensive Rats

**Figure 2:**
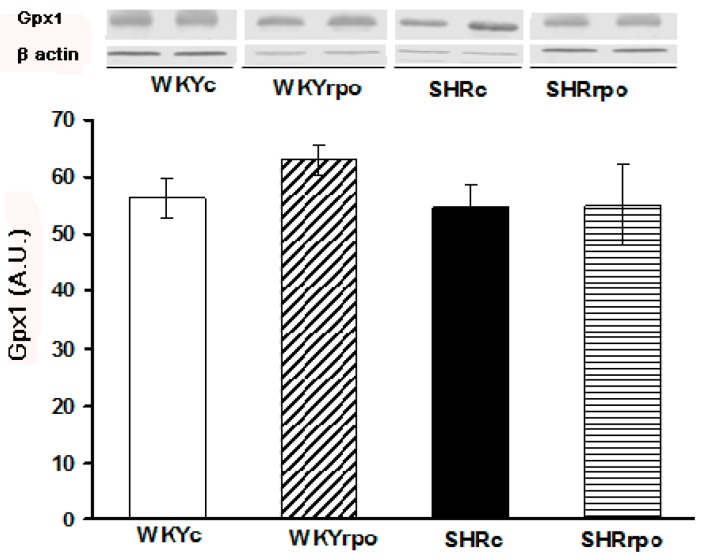
Effect of dietary red palm oil (RPO) supplementation on the protein expression of glutathione peroxidase 1 (Gpx1) in the left ventricle of normotensive and hypertensive rats. WKYc—Wistar Kyoto control rats; WKYrpo—WKY treated with RPO; SHRc—Spontaneously hypertensive rats; SHRrpo—SHR treated with RPO; Results are expressed as mean ± SEM; *n* = 5 in each group.

No significant differences were observed in myocardial Gpx1 protein expression between normotensive SHR and WKY (Figure 2). WKY supplemented by RPO showed the tendency to increase myocardial Gpx1 level while RPO did not affect Gpx1 expression in SHR hearts (Figure 2).

### 2.4. Effect of Dietary RPO Supplementation on the Nitric Oxide Synthase (NOS) Activity

**Figure 3:**
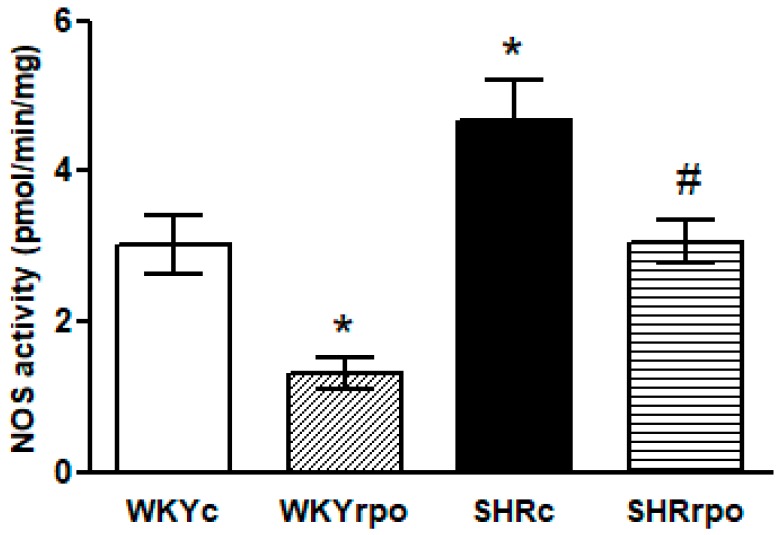
Effect of dietary red palm oil (RPO) on the nitric oxide synthase activity (NOS) in the left ventricle of normotensive and hypertensive rats. WKYc—Wistar Kyoto control rats; WKYrpo—WKY treated with RPO; SHRc—Spontaneously hypertensive rats; SHRrpo—SHR treated with RPO. Results are expressed as mean ± SEM; *n* = 5 in each group; * *p* < 0.05 versus control rats (WKYc); ^#^ *p* < 0.05 WKY/SHR versus treated with RPO.

Compared to control WKY hearts, nitric oxide synthase (NOS) activity was significantly increased in SHR hearts. Supplementation with RPO suppressed NOS activity in WKY and SHR (Figure 3).

### 2.5. Effect of RPO Supplementation on the Coronary Flow of Langendorff-Perfused Heart of Normotensive and Hypertensive Rats during Basal Condition and at Early-Phase of Post-Ischemic Reperfusion

**Figure 4:**
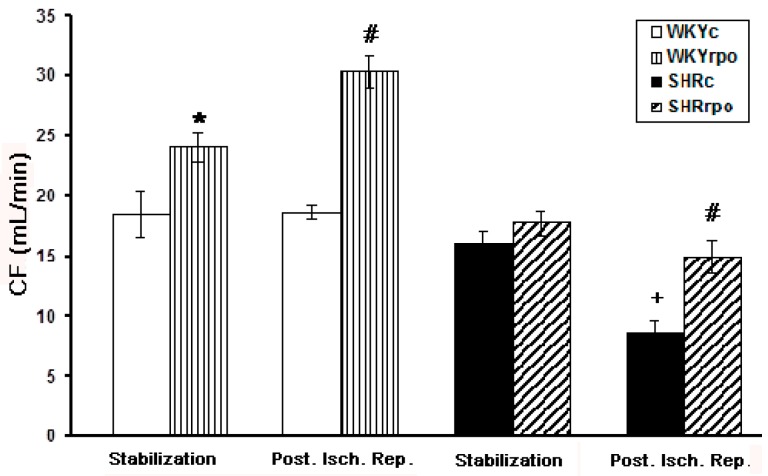
Effect of dietary red palm oil (RPO) supplementation on the coronary flow (CF) of WKY and SHR during stabilization and post ischemic reperfusion (Post. Isch. Rep). WKYc—Wistar Kyoto control rats; WKYrpo—WKY rats treated with RPO; SHRc—Spontaneously hypertensive rats; SHRrpo—SHR treated with RPO; Results are expressed as mean ± SEM; *n* = 6 in each group; * *p* < 0.05 versus control rats (WKYc); ^#^ *p* < 0.05 WKY/SHR versus treated with RPO; ^+^ *p* < 0.05 SHRc during stabilization versus SHRc during Post. Isch. Rep.

There were no significant differences in coronary flow (CF) between SHR versus WKY hearts registered at basal condition (Figure 4). RPO supplementation did not significantly affect basal CF in hypertensive rat hearts although there was a tendency to enhance it. On the other hand, RPO did result in a significant increase of CF in SHR during post-ischemic reperfusion comparing to untreated SHR group. In addition, RPO intake significantly enhanced CF during both basal and post-ischemic condition in WKY.

### 2.6. Effect of RPO Supplementation on the Heart Rate of Langendorff-Perfused Heart of Normotensive and Hypertensive Rats during Basal Condition and at Early-Phase of Post-Ischemic Reperfusion

**Figure 5:**
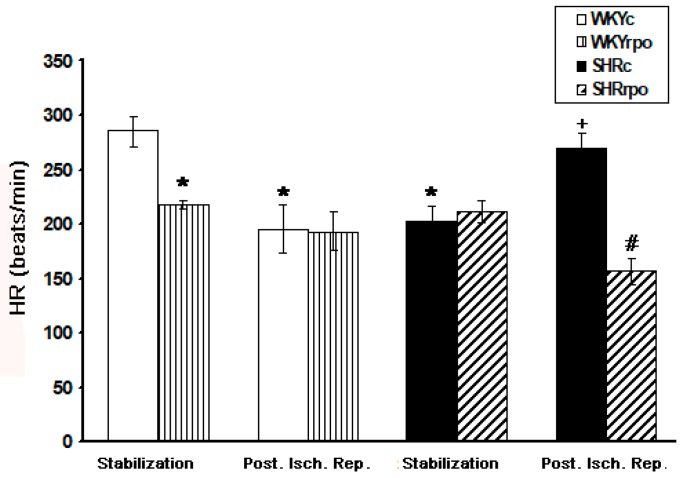
Effect of dietary red palm oil (RPO) supplementation on the heart rate of WKY and SHR during stabilization and post ischemic reperfusion (Post. Isch. Rep). WKYc—Wistar Kyoto control rats; WKYrpo—WKY treated with RPO; SHRc—Spontaneously hypertensive rats; SHRrpo—SHR treated with RPO. Results are expressed as mean ± SEM; *n* = 6 in each group; * *p* < 0.05 versus control rats (WKYc); ^#^ *p* < 0.05 WKY/SHR versus treated with RPO; ^+^ *p* < 0.05 SHRc during stabilization versus SHRc during Post. Isch. Rep.

Comparing to normotensive WKY rats the basal heart rate (HR) was significantly lower in SHR hearts and significantly increased in SHR hearts at early-phase of post-ischemic reperfusion (Figure 5). RPO supplementation did not affect basal HR in SHR while suppressed it in WKY hearts. Furthermore, RPO intake significantly reduced elevated HR at early-phase of post-ischemic reperfusion in SHR while did not change this parameter in WKY hearts (Figure 5).

### 2.7. Contraction and Relaxation Function Assessed by +dP/dt_max_ and −dP/dt_max_ in Langendorff-Perfused Heart of WKY and SHR during Basal Condition and at Early-Phase of Post-Ischemic Reperfusion

**Figure 6:**
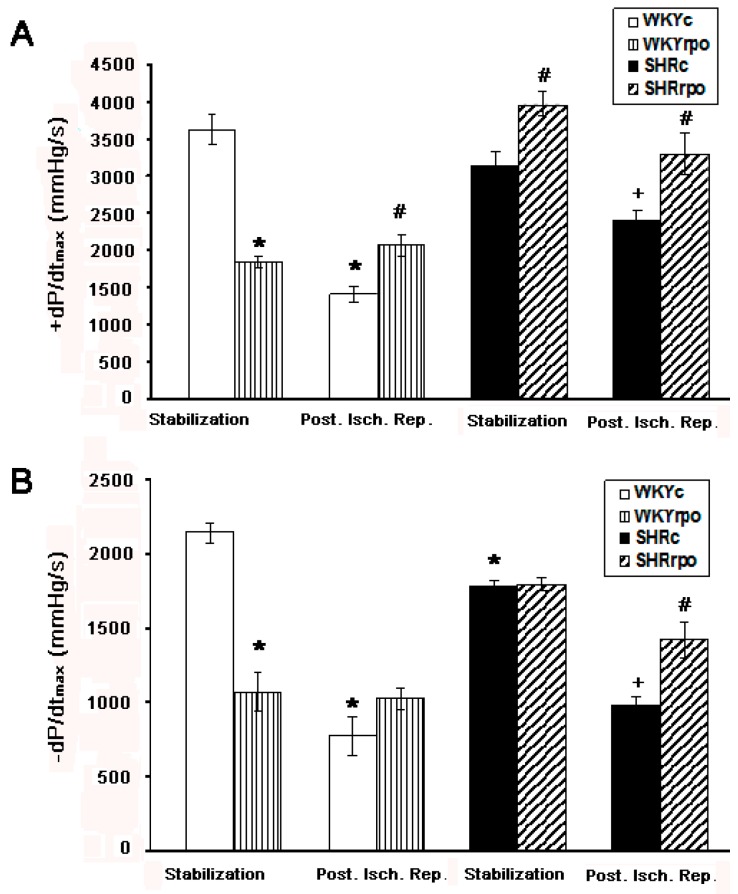
Effect of dietary red palm oil (RPO) supplementation on the contractile and relaxation function of left ventricle in WKY and SHR during stabilization and post ischemic reperfusion (Post. Isch. Rep). +dP/dt—the velocity of contraction (**A**); −dP/dt—the velocity of relaxation (**B**); WKYc—Wistar Kyoto control rats; WKYrpo—WKY treated with RPO; SHRc—Spontaneously hypertensive rats; SHRrpo—SHR treated with RPO. Results are expressed as mean ± SEM; *n* = 6 in each group; * *p* < 0.05 versus control rats (WKYc); ^#^ *p* < 0.05 WKY/SHR versus treated with RPO; ^+^ *p* < 0.05 SHRc during stabilization versus SHRc during Post. Isch. Rep.

Compared to normotensive WKY hearts the velocity of contraction of SHR was not altered unlike the velocity of relaxation, which was significantly decreased during basal condition (Figure 6A,B). Post-ischemic reperfusion resulted in significant deterioration of both contraction and relaxation function in WKY as well as SHR hearts. RPO supplementation significantly increased the velocity of contraction of SHR heart during basal condition (Figure 6A). Moreover. RPO significantly enhanced contraction and relaxation function during early-phase of post-ischemic reperfusion (Figure 6A,B). Supplementation of WKY with RPO resulted in suppression of contraction and relaxation function at basal condition (Figure 6A,B) but significantly attenuated deterioration of contractile function at early-phase of post-ischemic reperfusion (Figure 6A).

## 3. Discussion

This study investigated the effect of RPO supplementation on expression of selected myocardial endogenous antioxidant enzymes. i.e., SOD1. SOD2 and Gpx1 as well as NO production in the left ventricular tissue as potential mechanisms of protection of heart function in SHR.

RPO intake led to a significant reduction of elevated blood pressure in SHR by about 28%. Considering the crucial role of oxidative stress in development of hypertension [11,12], it seems that despite of presence of saturated fats in RPO, its antioxidant components may be beneficial in blood pressure regulation. Oxidized oils are thought to promote an increase in blood pressure likely due to aberrant endothelial function [8]. However, RPO did not affect increased of left ventricular weight. It suggests that short-lasting RPO intake may suppress blood pressure but do not reverse the hypertrophy.

Main findings of this study indicate that RPO intake normalize the expression of mitochondria related SOD2 that was significantly reduced in the hearts of SHR. Reduced expression or activity of SOD2 is a good indicator of mitochondrial oxidative stress and a sensitive indicator for myocardial oxidative stress [29,30,31]. Myocardial cells are highly specialized aerobic cells with a high number of mitochondria. The large amount of ROS produced by the myocardial mitochondria renders the heart more prone to oxidative stress. It is most likely that normalization of SOD2 expression in our study is linked with normalization of its activity resulting in attenuation of oxidative stress in SHR. RPO intake exhibited also the tendency to normalize lowered cytosolic SOD1 expression in SHR but the value did not rich significance. Mitochondrial SOD2 is an important antioxidant enzyme involved in dismutation of superoxide to H_2_O_2_ [32,33]. The latter is reduced to H_2_O by catalase and Gpx1 [17]. We found no differences in myocardial expression of specific Gpx1 isoform between SHR and WKY regardless of RPO supplementation. Accordingly, we can speculate that the myocardial activity of Gpx1 was sufficient to eliminate excess of H_2_O_2_ in conjunction with action of catalase. Taken together it appears that the cardio-protective effect of RPO in hypertensive rats is at least in part mediated by up-regulation of mitochondrial SOD2 protein expression with potential attenuation of oxidative stress. There is convincing scientific evidence showing the indispensable role of SOD2 in maintaining cardiovascular health. In this regard, Melov et al. [34] reported that mice exhibiting partial SOD2 deficiency had increased mitochondrial oxidative damage. It has also been shown that complete SOD2 deficiency in mice was associated with dilated cardiomyopathy and increased neonatal mortality [31]. On the other hand, overexpression of SOD2 decreased mitochondrial superoxide and restored NO bioavailability in hypertension [32]. SOD mimetic agents were associated with amelioration of oxidative stress and hypertension in SHR [35,36].The effect of RPO on myocardial SOD has previously been investigated using different pathological models but not in SHR. There was no effect on total SOD activity in hearts of hyperlipidemic rats exposed to ischaemia-reperfusion injury. However, the expression was not determined [24]. Another study demonstrated an increase of both mRNA and proteins levels of SOD1 as possible mechanisms by which RPO conferred cardioprotection in anthracycline-injured hearts [26]. This argues that the efficacy of RPO protection may be related to the extent of oxidative stress in various experimental models. Up-regulation of SOD2 found in SHR indicates that RPO increased the pool of available SOD2, which is a key mitochondrial antioxidant. Hence, RPO enhanced intrinsic endogenous antioxidant defense system in the heart affected by hypertension. Thus, interventions aimed at combating oxidative stress should be specifically targeted at the primary site of ROS production such as the mitochondria in order for them to be effective. Therefore, targeting the mitochondrial antioxidant enzymes, which may lead to alleviation of mitochondrial oxidative stress, may be considered as a crucial therapeutic target. This approach is supported by the studies showing that cardiac arrhythmias could be prevented by mitochondria-targeted antioxidants rather than general antioxidants [37]. It fits with our previous findings indicating that SHR benefit from RPO intake because of its apparent anti-arrhythmic effect. This can be attributed to the protection of myocardial electrical coupling protein Cx43 [27] impaired by oxidative stress [8].

SODs play a critical role also in inhibiting oxidative inactivation of NO and preventing peroxynitrite formation, thereby mitochondrial and endothelial dysfunction [17]. Superoxide and other reactive oxygen species can avidly react with and inactivate NO. We found that NOS activity was increased in left ventricle of SHR as compared to WKY rat hearts. It is consistent with previous results obtained in young and old SHR [38]. Enhancement of myocardial NOS activity might be attributed to the up-regulation of inducible NOS (iNOS or NOS1) protein expression that was demonstrated in the heart of SHR [39,40,41]. Enhanced NO inactivation can be also indicator of the incidence of severe hypertension. [41]. This can, in turn cause a compensatory up-regulation of NOS isotype expression [41]. Of note, RPO supplementation resulted in normalization of NOS activity in SHR hearts. Likewise, NOS activity was normalized due to intake of omega-3 polyunsaturated fatty acids (exhibiting antioxidant properties) that also reduced blood pressure in SHR [38]. Moreover, antioxidant therapy with lazaroid alleviated hypertension and reversed the compensatory up-regulation of NOS isotypes in SHR [41]. These findings support the implication of oxidative stress in the genesis and/or maintenance of hypertension and benefit of targeted antioxidants. However, it should be noted that RPO intake suppressed NOS activity in WKY hearts as well. Therefore, we assume that the treatment doses of RPO, especially antioxidants, are probably too high for healthy rats and may result even in undesirable effects [42].

Furthermore, our original findings indicate that RPO related attenuation of hypertension-induced abnormalities in myocardial SOD2 and NOS were associated with the protection of heart function during post-ischemic reperfusion. It was demonstrated by significant enhancement of coronary flow that was reduced (by about 50%) due to post-ischemic reperfusion and by significant suppression of elevated heart rate. Importantly, RPO intake significantly improved contraction and relaxation of the left ventricle that was dramatically deteriorated at early-phase of post-ischemic reperfusion. Taking into account excessive production of ROS during post-ischemic reperfusion that deteriorates heart function, our findings are in favour of RPO antioxidant effects. The latter may also explain clear cut anti-arrhythmic effects of RPO in arrhythmia prone SHR as well as better ability of RPO treated SHR hearts to restore sinus rhythm after induction of arrhythmia [27]. Preserving the reperfused myocardium is an important task in clinical cardiology to reduce morbidity and mortality after myocardial infarction. It is of interest to researchers that new approaches include those that target mitochondrial function and energetic as well as prevention of no-reflow phenomenon [43]. Besides prevention of excessive ROS, one of the possible mechanisms by which RPO might confer cardiovascular protection during ischemia/reperfusion is via increased phosphorylation of Akt kinase [19,21]. This activation might be especially important in the heart of SHR where the basal NOS activity was significantly elevated [44].

Interestingly, RPO intake resulted in significant decrease of heart rate as well as indexes of contractility and relaxations. +dP/dt_max_ and –dP/dt_max_ in normotensive WKY. It suggests that “therapeutic” dose of RPO is cardio-depressive on healthy heart. On the other hand, beneficial effect of RPO was demonstrated during post-ischemic reperfusion of normotensive WKY likewise hypertensive rat hearts.

## 4. Materials and Methods

### 4.1. Animal Care and Ethical Considerations

We have used in our experiments animals approved by the State Veterinary Administration of the Slovak Republic, legislation No. 377/2012 (14 November 2012) and in accordance with the European Union Council Directive 86/609/EEC about the protection of animals used for experimental and other scientific purposes [27]. Male, three-month-old SHR and non-hypertensive WKY fed a standard rat chow plus RPO (200 μL/day, Carotino Sdn. Bhd., Johor Bahru, Malaysia) for 5 weeks were used.

### 4.2. Animal Monitoring and Tissue Sampling

Tail-cuff plethysmography was used for the blood pressure measurement (Statham Pressure Transducer P23XL, Hugo Sachs, March-Hugstetten, Germany). It was registered as well as body weight at the beginning and the end of the experiments. Fasting blood glucose was measured by using blood glucose test meter. The total cholesterol was determined in the blood serum. After rat anesthesia, the hearts were rapidly removed and placed into ice-cold saline what lead into stopping heartbeat. Total heart and left ventricle weigh were registered. Analysis of myocardial antioxidant enzymes and total NOS activity was determined immediately in fresh tissue of the left ventricle [27].

### 4.3. Experimental Protocol of Isolated Langendorff-Perfused Heart

**Figure 7:**
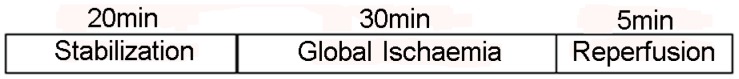
Study design showing perfusion protocol.

Langendorff perfused system with oxygenated Krebs-Henseleit solution was used for perfusion of rat hearts which were cannulated via aorta at constant pressure of 80 mmHg and temperature of 37 °C. Intraventricular balloon was used for isovolumetrically measurement of the left ventricular pressure (LVP) [45]. LVP, heart rate and the coronary flow (CF) were continuously monitored during experiment for the evaluation of heart function. LVP and the positive and negative first derivatives of LVP, +dP/dt (mmHg/s) and −dP/dt (mmHg/s) were monitored as indicators of contractile heart function in regard to the rate of increase (due to contraction) and rate of decrease (due to relaxation) of intraventricular pressure, respectively [45]. Upon 20 min of equilibration, the heart was subjected to 30 min global ischemia followed by 5 min reperfusion to examine early reperfusion-induced changes in the heart function (Figure 7).

### 4.4. Measurement of NOS Activity

Left ventricle tissue homogenates were used for NO synthase activity measurement. Approximately 200 mg/mL of heart tissue was homogenized in ice cold homogenisation buffer containing 1% protease inhibitor cocktail (104 mmol/L 4-(2-aminoethyl) benzenesulfonyl fluoride, 80 μmol/L aprotinin, 4 mmol/L bestatin, 1.4 mmol/L E-64, 2 mmol/L leupeptin, 1.5 mmol/L pepstatin A, purchased from Sigma-Aldrich, St. Louis, MO, USA) in 0.05 mol/L Tris-HCl, pH 7.4, by Ultra-Turrax homogeniser and subsequently centrifuged (15 min/9500× *g*) at 4 °C. Total NO synthase activity was obtain from supernatants samples by conversion of [3H]-l-arginine (MP Biomedicals, Santa Ana, CA, USA, 50 Ci/mmol) to [3H]-l-citrulline [46,47] and expressed as pmol/min/mg of tissue proteins as determined by the Lowry method [48].

### 4.5. Analysis of Myocardial SOD1, SOD2 and Gpx1 Expression Using Western Blot Analysis

Analysis of basal myocardial antioxidant protein expression levels was carried with the use of western blot protein analysis. Myocardial left ventricular tissue was solubilized in a lysis buffer (in mM: Tris 20, *p*-nitrophenylphosphate 20, EGTA 1, NaF 50, sodium orthovanadate 0.1, phenylmethyl sulfonyl fluoride (PMSF) 1, dithiothreitol (DTT) 1, aprotinin 10 μg/mL). Samples were diluted in Laemmli sample buffer and boiled for 5 min. Approximately 50 μg of proteins per line were loaded into 12.5% acrylamide gels and separated by SDS PAGE (Bio-RAD Mini Protein Tetra cell 552BR, Hercules, CA, USA). Proteins from the gels were transferred into a PVDF membrane (Immobilon-P, Millipore, Burlington, MA, USA) and blocked with 5% fat-free milk in Tris-buffered saline with 0.1% Tween 20 (TBST). For SODs and Gpx determination were membranes incubated with specific primary antibodies (SOD1 / Gpx1 diluted 1:1000 and SOD2 diluted 1:2000, overnight), washed in TBST (5 × 3 min) and incubated for one hour with the secondary antibody conjugated with alkaline-phosphotase. For proteins visualization were membranes topped with a chromogenic substrate (ProteinDetector^TM^, Western Blot Kit, BCIP/NBT System, KPL Inc., Ocracoke, NC, USA). Protein loading was normalized with β actin to ensured equal protein loading for different lanes. Antibodies were purchased from Cell Signalling (Danvers, MA, USA) and Abcam (Cambridge, UK). Other chemicals were obtained from Bio-RAD and Sigma (St. Louis, MO, USA) [21].

### 4.6. Statistical Analysis

The data are expressed as means ± SEM. A one way ANOVA and Bonferroni post *hoc* test were used to determine statistical significance between means in multiple groups. While the differences between groups were determined using an unpaired Student’s *t*-test. Values were considered to differ significantly when *p* < 0.05.

## 5. Conclusions

In light of the evidence presented in this study we propose that the increased mitochondrial SOD2 protein expression in the heart could be considered as possible mechanisms for RPO induced cardiovascular protection in SHR. The increase in SOD2 expression could result in reduced oxidative stress and consequently abolish of post-ischemic reperfusion related heart dysfunction. To our knowledge this is the first evidence implicating the beneficial effect of RPO through augmentation of SOD2 and improved heart function at early-phase of post-ischemic reperfusion in SHR heart.

## Figures and Tables

**Table 1:** General characteristics of the male of normotensive and hypertensive rats without and with red palm oil intake.

Parameter	WKYc	WKYrpo	SHRc	SHRrpo
BP (mmHg)	114.42 ± 11.53	120.71 ± 14.64	185.73 ± 12.52 *	134.72 ± 10.94 ^#^
BW (g)	400.73 ± 20.22	378.21 ± 31.22	311.44 ± 18.11 *	332.63 ± 20.94
HW (g)	1.33 ± 0.08	1.34 ± 0.09	1.51 ± 0.06	1.47 ± 0.04
LVW (g)	0.72 ± 0.03	0.74 ± 0.06	0.95 ± 0.03 *	0.89 ± 0.07
BG (mg/dL)	5.49 ± 0.83	4.98 ± 0.91	6.12 ± 0.82	5.78 ± 0.73
CHOL (mmol/L)	2.43 ± 0.42	2.38 ± 0.33	2.78 ± 0.62	2.74 ± 0.22

BP—Blood pressure; BW—Body weight; HW—Heart weight; LVW—Left ventricular weight; BG—Blood glucose; CHOL—Cholesterol; WKYc—Wistar Kyoto control rats; WKYrpo—WKY treated with RPO; SHRc—Spontaneously hypertensive rats; SHRrpo—SHR treated with RPO; Data are presented as mean ± SEM; *n* = 13 in each group; * *p* < 0.05 versus control rats (WKYc); ^#^ *p* < 0.05 WKY/SHR versus treated with red palm oil (RPO).
